# CEST MRI Affirms HIV-1-Associated Neurometabolic Impairments in a Humanized Mouse Model

**DOI:** 10.21203/rs.3.rs-6821484/v1

**Published:** 2025-07-02

**Authors:** Gabriel C. Gauthier, Micah Summerlin, Balasrinivasa R. Sajja, Mariano G. Uberti, Emma G. Foster, Manjeet Kumar, Matthew Thiele, Santhi Gorantla, Aditya N. Bade, Yutong Liu

**Affiliations:** University of Nebraska Medical Center; University of Nebraska Medical Center; University of Nebraska Medical Center; University of Nebraska Medical Center; University of Nebraska Medical Center; University of Nebraska Medical Center; University of Nebraska Medical Center; University of Nebraska Medical Center; University of Nebraska Medical Center; University of Nebraska Medical Center

**Keywords:** Human immunodeficiency virus (HIV), anti-retroviral therapy (ART), HIV-associated neurocognitive disorders (HAND), magnetic resonance imaging (MRI), chemical exchange saturation transfer (CEST), humanized mice

## Abstract

**Purpose:**

Human immunodeficiency virus 1 (HIV-1)-associated neurocognitive disorders (HAND) persist in people living with HIV-1 (PLWH) despite antiretroviral therapy (ART), driven by unresolved neuroinflammation and metabolic dysfunction. This study evaluates chemical exchange saturation transfer (CEST) magnetic resonance imaging (MRI) detection of HIV-1-induced neurometabolic impairments and ART-mediated improvements in a humanized mouse model.

**Methods:**

HIV-1-infected CD34-NSG mice (n = 14) underwent CEST MRI to quantify metabolic profiles in the cortex, hippocampus, hypothalamus, piriform cortex, and thalamus at three timepoints: pre-infection (Week 0), 6 weeks-post-infection (WPI), and after 6 weeks of ART- or vehicle-treatment (12 WPI). CEST contrasts were analyzed at 2 ppm (creatine), 3 ppm (glutamate), and − 3.5 ppm (nuclear Overhauser effect, NOE). Neuroinflammation and infection were confirmed using immunohistology and qPCR.

**Results:**

At 6 WPI, HIV-1-infection reduced creatine in the cortex (p = 0.0006) and hippocampus (p = 0.01), and elevated NOE in the cortex (p = 0.001). At 12 WPI, vehicle-treated HIV-infected mice exhibited significantly decreased glutamate in the cortex (p = 0.004), hippocampus (p < 0.0001), and piriform cortex (p = 0.002); ART-treatment restored these levels in the cortex and hippocampus. Further, vehicle-treated mice exhibited decreased creatine in the cortex (p = 0.0002), hippocampus (p = 0.0003), piriform cortex (p = 0.0009), and thalamus (p = 0.006); ART-treatment restored these levels in the hippocampus, piriform cortex, and thalamus. Finally, vehicle-treated mice exhibited increased NOE in the cortex (p = 0.002) and thalamus (p = 0.003), but this was not restored by ART. CEST findings were supported by reductions in HIV-1 p24 + cells and neuroinflammatory markers in ART-treated brains.

**Discussion:**

CEST MRI detects region-specific HIV-1-induced neurometabolic alterations and ART-mediated restorations. This work establishes CEST MRI as a translational potential, non-invasive technique for monitoring HAND pathology and therapeutic efficacy.

## INTRODUCTION

Although antiretroviral therapy (ART) has transformed human immunodeficiency virus 1 (HIV-1) infection into a manageable chronic illness, treatment outcomes remain highly variable, necessitating further improvements[1–6]. Effective viral suppression requires lifelong adherence, posing challenges for many, due to personal, social, and financial barriers[1, 3–5, 2]. Even with consistent access to care and undetectable viral loads, a significant portion of people living with HIV-1 (PLWH) experience HIV-1-associated neurocognitive disorders (HAND). HAND encompasses a spectrum of neurocognitive impairments, including asymptomatic neurocognitive impairment (ANI), mild neurocognitive disorder (MND), and HIV-1-associated dementia (HAD)[7–15]. Any of these impairments can adversely affect daily functioning and quality of life[7, 10–12, 8, 9, 13, 16, 14, 15].

HAND is closely linked to persistent neuroinflammation or neuroimmune dysfunction, driven by viral reservoirs in the central nervous system (CNS) [7, 10–12, 8, 9, 13, 16, 14, 15]. The blood-brain barrier (BBB) limits ART penetration into the CNS[17–22], resulting in suboptimal drug concentrations. This leads to the formation of latent viral reservoirs in infected microglia, perivascular macrophages, and astrocytes, sustaining low-levels of viral replication and subsequent neuroinflammation and neuronal injury [23–26, 7, 14]. Thus, these neuropathologies collectively perpetuate HAND, even while undergoing ART [7, 12, 8, 13, 14]. Furthermore, some antiretroviral drugs (ARVs) have been implicated in exacerbating glial dysfunction and neuronal impairments[7, 10, 12, 8, 9, 13, 16, 14, 4–6, 2]. Despite significant efforts to study effects of HIV-1 and ART on CNS homeostasis, there remains limited understanding of effects on CNS metabolites.

This study employs chemical exchange saturation transfer (CEST) magnetic resonance imaging (MRI) approach to investigate the impact of HIV-1_ADA_ infection and ART on brain metabolic profiles in a humanized mouse model. CEST MRI is an advanced molecular imaging technique that enables non-invasive detection of biomolecules, even at low concentrations, through proton exchange processes[27–31, 16, 32–36]. Specifically, CEST MRI exploits the chemical exchange between protons of biomolecules (e.g., metabolites, neurotransmitters) and bulk water protons. By applying a frequency-selective saturation pulse to protons in specific solute molecules, solute proton-exchange with water reduces the measurable water signal, creating a contrast proportional to the concentration of the solute molecule. This approach allows CEST MRI to indirectly visualize metabolites, including glutamate at 3 ppm [37, 31, 38–43], creatine at 2 ppm [44–49], and macromolecules (e.g., mobile proteins, lipids) via relayed nuclear Overhauser effect (NOE) at −3.5 ppm [50–52] with higher sensitivity and spatial resolution than conventional magnetic resonance spectroscopy (MRS). Thus, this study leverages CEST MRI to map regional neurometabolic disruptions (glutamate, creatine) and macromolecular changes (NOE) in a humanized mouse model of HIV-1 infection, with and without ART. Overall, we aim to identify imaging biomarkers for detecting persistence and progression of HAND-linked pathobiology.

## MATERIALS AND METHODS

### Generation of Humanized Mice and HIV-1 Infection

All animal studies included in this manuscript were approved by the University of Nebraska Medical Center (UNMC) Institutional Animal Care and Use Committee (IACUC) in accordance with the standards of the Guide for the Care and Use of Laboratory Animals (National Research Council of the National Academies, 2011). Male CD34-NSG humanized mice (n = 14) were used to evaluate the effects of HIV-1_ADA_ infection and ART on CNS metabolic profiles. These mice were designed to mimic human immune responses and study HIV-1-linked neuropathology[53, 54]. Detailed procedures for generating humanized mice is described in previous studies[53]. Briefly, NSG (NOD.Cg-Prkdc^scid^Il2rgt^m1Wjl^/SzJ) mice were bred and maintained at UNMC under pathogen-free conditions in accordance with the ethical guidelines set forth by the National Institutes of Health (NIH) for the care of laboratory animals. Human CD34 + hematopoietic stem progenitor cells (HSPCs) cells were isolated from human cord blood obtained from healthy full-term newborns (Department of Gynecology and Obstetrics, UNMC). Isolation of CD34 + cells was completed using immune-magnetic beads according to the manufacturer’s instructions (CD34 + selection kit; Miltenyi Biotec Inc., Gaithersburg, MD). The purity (> 90%) and numbers of CD34 + cells were confirmed using flow cytometry. On post-natal day 1–3, neonates were irradiated with 1 Gy using an RS-2000 X-ray Irradiator (Rad Source Technologies). 4 hours after irradiation, neonates were intrahepatically (i.h.) injected with 10^5^ CD34 + HSPCs in 20 μL phosphate-buffered saline (PBS) using a 30-gauge needle. Human cell engraftment was confirmed by collecting peripheral blood by cheek-bleeding and performing flow cytometry. At 20–22 weeks of age, humanized mice were selected for the study based on the total human CD45 + cell count (> 20%). For HIV-1 infection, humanized mice were intraperitoneally (i.p.) injected with HIV-1_ADA_ at 10^4^ tissue culture infectious dose_50_ (TCID_50_).

### Experimental Design

**Figure 1:**
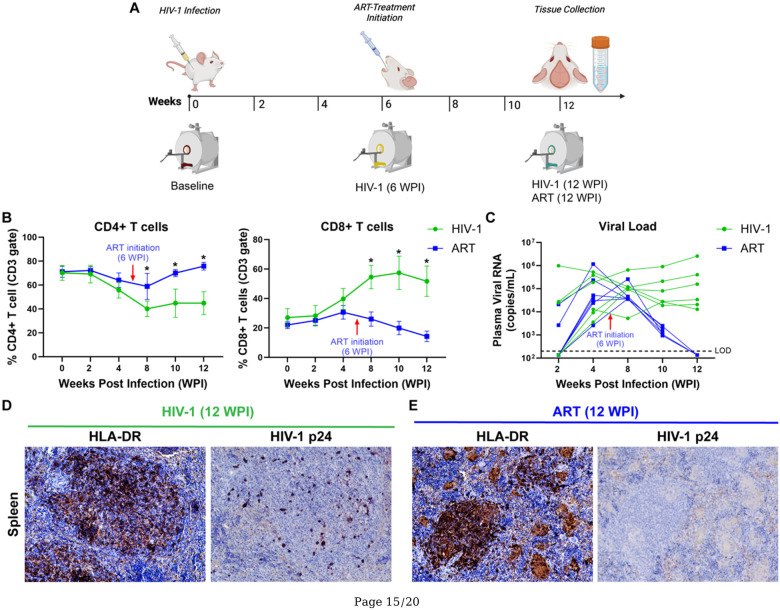
(**A**) Experimental design: Humanized mice underwent multimodal MRI scans prior to HIV-1 infection at baseline (0 WPI) followed by biweekly cheek bleeding starting at 2 WPI to assess plasma viral load and T cell counts. A second MRI was conducted at 6 WPI, prior to initiating daily ART treatment, with a final MRI scan at 12 WPI. Euthanasia was conducted following MRI for tissue collection. (**B**) Flow cytometry results: CD4+ and CD8+ T cell counts were tracked post-infection and following ART or vehicle treatment. (**C**) Plasma viral RNA: dynamics of plasma viral load post-infection and after treatment was measured using the automated COBAS Ampliprep system. Immunohistological analysis of spleen at 12 WPI in (**D**) vehicle- and (**E**) ART-treated mice.

Experimental design is illustrated in Fig. 1A. Baseline imaging including T_2_-weighted anatomical scans and CEST MRI was conducted on humanized mice before HIV-1 infection (Week 0). Immediately following the baseline scan, the mice were infected with HIV-1 as described above. At 6 weeks-post-infection (WPI), infection was confirmed in all animals by measuring plasma viral load. Immediately after viral confirmation, animals were scanned again to determine short-term effects of infection. Immediately after the 6 WPI scan, HIV-1-infected mice were randomly divided into two treatment groups: ART (n = 7) and vehicle (Control; n = 7). Animals were treated daily with ART or vehicle for 6 weeks, with treatment duration covering 6 WPI to 12 WPI. Imaging was repeated once more at 12 WPI to assess effects of long-term infection and ART benefits. Due to loss of animals during the study period and observed distress in some animals, only five vehicle-treated mice and four ART-treated mice successfully underwent 12 WPI MRI scans. Mice were euthanized immediately after final imaging. Brain and spleen tissue were collected for immunohistological analyses. Biweekly peripheral blood was collected from the submandibular vein (cheek bleed) to measure temporal plasma viral RNA and CD4+/CD8 + T-cell counts as biomarkers of HIV-1 infection (Fig. 1A).

### ART Administration

HIV-1 infected animals were administered every day with ART or vehicle by oral gavage, from 6 WPI to 12 WPI (Fig. 1A). The ART regimen was comprised of tenofovir disoproxil (123 mg/kg; mouse weight), lamivudine (123 mg/kg; mouse weight), and dolutegravir (20.5 mg/kg; mouse weight) in combination, also known as TLD. The dosage corresponds to double the human equivalent dose (HED) of TLD [55, 56]. The vehicle [dimethylsulfoxide:Solutol^®^:50mM N-methylglucamine in 3% mannitol (1:1:8, v:w:v)][57], utilized to create suspension of the drugs, was administrated to controls at the same volume and scheme as the TLD administration.

### Measurements of Plasma Viral Load and T Cell Counts

Mice peripheral blood samples were taken biweekly from the submandibular vein (cheek bleed) by using 5 mm lancets (MEDIpoint, Inc., Mineola, NY) and collected in EDTA coated tubes. Quantitative viral load measurements were conducted using the automated COBAS Ampliprep system v2.0/Taqman-48 system (Roche Molecular Diagnostics, Basel, Switzerland) as described previously[58–60]. The limit of detection for viral load measurement is 200 viral RNA copies/mL according to the dilution factor[58]. In parallel, peripheral blood human-immune-cell profile was examined by flow cytometry. After collection of excess plasma, 25 μL of whole blood suspensions were incubated with a combination of human monoclonal antibodies for CD45, CD3, CD4, and CD8 markers for 30 minutes [53, 58, 60]. Red blood cells (RBCs) were lysed with FACS lysing solution (BD Biosciences, Franklin Lakes, NJ, USA) and finally stained cells were fixed with 2% paraformaldehyde (PFA). Flow cytometry was carried out with a standard procedure using the LSR-II FACS analyzer[53]. Targeted-cell-specific populations were analyzed using FlowJo v10.5 (BD Pharmingen, San Diego, CA, USA) and percentages of total number of gated lymphocytes were expressed as results.

### Magnetic Resonance Imaging (MRI)

MRI was performed on a 7T Bruker PharmaScan system using a Bruker quadrature mouse head coil for all scans. Mice were anesthetized with 1–3% isoflurane during scanning, and their breathing was monitored throughout.

### T_2_-Weighted MRI

A RARE sequence was used to acquire T_2_-weighted images on axial planes with the following parameters: TE = 48 ms, TR = 4.2 s, 4 averages, RARE factor = 8, number of slices = 21, slice thickness = 0.5 mm, FOV = 20 mm × 20 mm, and matrix = 256 × 192.

### CEST MRI

CEST MRI data were acquired using a RARE sequence with a saturation RF amplitude of 2 *μ* T, a duration of 2 s, and a frequency offset range from − 5 to 5 ppm. An offset step = 0.2 ppm was used in the ranges of [−5 ppm, −4 ppm] and [4 ppm, 5 ppm], while an offset step = 0.1 ppm was used in [−4 ppm, 4 ppm]; this non-uniform frequency sampling allowed us to adequately observe all relevant offsets, while prioritizing accuracy within the central range where CEST contrasts at 2 and 3 ppm and NOE at −3.5 ppm are observed. The scan included 2 slices that were positioned to include cortex, hippocampus, hypothalamus, piriform cortex, and thalamus, with FOV = 20 × 20 mm^2^, slice thickness = 0.5 mm, and matrix = 256 × 256. The main magnetic field (B0) correction was performed using WASSR[61]. CEST data was analyzed using 5-pool Lorentzian fitting for the measurements of contrasts at 2 ppm, 3 ppm and − 3.5 ppm, −1 ppm, and 0 ppm. The metabolites underlying the 2 ppm and 3 ppm contrasts are creatine and glutamate, respectively. The contrast at −3.5 ppm is associated with NOE of macromolecules, and the contrast at −1 ppm results from the magnetization transfer (MT) from semi-solid macromolecules. Direct water suppression is fitted at 0 ppm. Each CEST contrast was estimated by calculating the area under the curve (AUC) of the Lorentzian function.

### Immunohistology

At 12 WPI, mice were humanely euthanized immediately after imaging, and brains and spleen were collected. Tissues were fixed in 4% PFA overnight. Later fixed tissues were processed in the Epredia^™^ STP 120 Spin Tissue Processor (Epredia^™^, Thermo Fisher Scientific, Waltham, MA, USA) using the standard overnight protocol, followed by embedding in paraffin blocks. Tissue sections of 5 μm in thickness were collected and stained with mouse monoclonal antibodies for HLA-DQ/DR/DP (Novus Biologicals, LLC, Centennial,), or HIV-1 p24 (Santa Cruz Biotechnology, Inc., Dallas, TX), and rabbit polyclonal antibodies for glial fibrillary acidic protein (GFAP) (Dako, Carpinteria, CA), or ionized calcium binding adaptor molecule − 1 (Iba-1) (FUJIFILM Wako Pure Chemical Corporation, Osaka, Japan). The polymer-based HRP-conjugated anti-mouse and anti-rabbit Dako EnVision systems were used as secondary detection reagents. 3,3’-diaminbenzidine (DAB, Dako, Carpinteria, CA) was used as a chromogen. All paraffin-embedded sections were counterstained with Mayer’s hematoxylin. Images were captured with a 20× objective using Nuance EX multispectral imaging system (CRi, Wobum, MA, USA)[53].

### RT-qPCR Assay

Total RNA was isolated from brain cortex tissues using TRIzol reagent (Invitrogen, Waltham, MA, USA). The RNA concentration was measured using nanodrop spectrophotometer (Thermo Fisher Scientific, Waltham, MA, USA). Further, cDNAs were synthesized from RNA using verso cDNA synthesis kits (Thermo Fisher Scientific), as per the manufacturer’s protocol. The synthesized cDNA was used as template for reverse transcription-quantitative polymerase chain reaction (RT-qPCR). Real-time PCR amplification was performed on the Quant Studio^™^ 5 System using SYBR green master mix (Applied Biosystems, Waltham, MA, USA). The HIV-1 RNAs were quantified using the following primers. For HIV-1 Gag, FP 5′-ATCTGGCCTGGTGCAATAGG-3 and RP 5′-ACATCAAGCAGCCATGCAAAAT-3; for GAPDH, FP 5-TGAGCAAGAGAGGCCCTATC-3 and RP 5-AGGCCCCTCCTGTTATTATG-3. The RT-qPCR was performed under the following conditions: 50°C for 2 min, 95°C for 10 min, 45 cycles of 95°C for 15 s and 60°C for 1 min. The fold change of the target gene (HIV-1 Gag) expression was calculated using ΔΔC_T_ method after normalization with an endogenous mouse GAPDH transcript expression of total RNA.

### Statistical Analysis

Comparisons between all groups were conducted using ordinary one-way ANOVA. Post-hoc comparisons between pre-infection baseline data and data at 6 WPI were conducted using two-tailed Student’s t-tests to evaluate the effects of short-term infection on imaging results. Post-hoc comparisons between the baseline and HIV-infected, vehicle-treated mice at 12 WPI represent the effects of prolonged infection and were conducted using two-tailed t-tests with Welch’s correction to account for the change in sample size. Post-hoc two-tailed t-tests were conducted to assess the impact of ART treatment by comparing the HIV-infected, ART-treated mice with the HIV-infected, vehicle-treated mice. Data were processed using GraphPad Prism. Data were expressed as mean ± standard error of the mean (SEM) with a minimum of 3 biological replicates. Statistical significance was denoted as ^#^p < 0.1, *p < 0.05, **p < 0.01, ***p < 0.001, ****p < 0.0001.

## RESULTS

### Viral and Immune Profile of HIV-1 Infected Humanized Mice

At 20–22 weeks of age, humanized mice (n = 14) were infected with HIV-1_ADA_ (10^4^ TCID_50_) at Week 0 (Fig. 1A). Productive infection was confirmed by biweekly assessments of plasma viral load and immune parameters via flow cytometry through 12 WPI (Fig. 1B–C). After confirmation of HIV-1 infection at 6 WPI (Fig. 1B–C), mice were randomly assigned to two treatment groups: vehicle-treated (control, n = 7) and ART-treated (n = 7). Beginning at 6 WPI, mice were administered daily with either ART or vehicle by oral gavage until 12 WPI. The ART regimen, known as TLD, included tenofovir disoproxil fumarate (TDF; 123 mg/kg), lamivudine (3TC; 123 mg/kg), and dolutegravir (DTG; 20.5 mg/kg). Among available ART regimens, TLD was chosen due to its wide usage in resource-limited countries (RLCs)[62–68]. Dosages were selected to attain twice the clinically relevant therapeutic levels[56]. For the control group, HIV-1 infected mice were treated with the vehicle used to prepare ART suspension for oral administration. The volume and administration strategy for vehicle was kept the same as TLD administration. MRI scans were performed before HIV-1 infection to obtain baseline data (Week 0). After confirmations of productive HIV-1 infection, all animals were scanned with same MRI scheme at 6 WPI prior to beginning of ART- or vehicle treatment. Finally, at the termination of the study (12 WPI), animals from both groups were scanned and humanely euthanized for the collection of blood and tissues for pathobiological assessments (Fig. 1A).

Firstly, flow cytometry was performed to determine reconstitution of peripheral human immune cells (CD45+, CD3+, CD4+, CD8+) and how HIV-1 or ART treatment affected them. The temporal changes of CD4 + and CD8 + T cells in animals of both groups are shown in Fig. 1B. Temporal assessments of blood CD4 + or CD8 + T cells were completed from the total human CD45 + and CD3 + gates for mice of vehicle-treated and ART-treated groups (Fig. 1B and Supplemental Fig. 1). The steady decline in CD4 + T cells was readily seen in all mice following HIV-1 infection. Levels of CD4 + T cells declined from 70% at baseline to 56% by 4 WPI in mice from vehicle-treated group (green color) and from 71% at baseline to 64% by 4 WPI in mice from ART-treated group (blue color). After the initiation of treatment at 6 WPI, in the vehicle-treated mice, CD4 + cells continued to decline, reaching to 45% at 12 WPI. In contrast, CD4 + cell counts in ART-treated mice recovered from 64% at 4 WPI to 76% at 12 WPI. During the treatment period, CD4 + T cells were significantly higher in ART-treated mice compared to vehicle-treated mice at 8 WPI (p = 0.009), 10 WPI (p = 0.04) and 12 WPI (p = 0.02). In parallel to a decline in CD4 + T cells, concomitant increases in CD8 + T cells were noted following HIV-1 infection in all mice (Fig. 1B). Levels of CD8 + T cells increased from 27% at baseline to 40% by 4 WPI in mice from the vehicle-treated group and from 22% at baseline to 31% by 4 WPI in mice from ART-treated group. Following initiation of treatments at 6 WPI, CD8 + T cells in the vehicle-treated mice continued to increase, reaching 52% by 12 WPI. Conversely, in the ART-treated mice, CD8 + T cells declined gradually, dropping from 31% at 4 WPI to 14% at 12 WPI. During the treatment period, CD8 + T cells were significantly lower in ART-treated mice than in vehicle-treated mice at 8, 10 and 12 WPI (p = 0.01, 0.02, and 0.01, respectively). Further, to confirm productive viral infection, temporal plasma viral RNA levels were measured (Fig. 1C). Pre-treatment plasma viral loads peaked around 4 weeks in all mice following infection. In vehicle-treated mice, plasma viral loads ranged between 3.56 × 10^3^ and 5.23 × 10^5^ RNA copies/mL, while for ART-treatment mice, values ranged between 2.60 × 10^3^ and 1.16 × 10^6^ RNA copies/mL. Following six weeks of vehicle-treatment, sustained viral replication was observed in control mice at 12 WPI (1.29 × 10^4^ to 2.57 × 10^6^). However, in ART-treated mice, plasma viral loads were below the limit of detection (LOD) (Fig. 1C).

Further, immunohistology assessments were completed to determine the human cell reconstitution and the level of HIV-1 infection in the spleen tissues of vehicle- and ART-treated mice (Fig. 1D–E). At 12 WPI, human cell reconstitution in spleen tissue was determined by immunohistochemical staining with human HLA-DR antibodies. Staining confirmed human cell penetration into the white and red pulp and germinal centers of spleen from mice of both groups (Fig. 1D–E). In parallel, HIV-1 infection was confirmed by immunohistochemical staining with HIV-1p24 antibodies. Robust HIV-1 infection was observed in vehicle-treated mice as shown by large numbers of stained cells (Fig. 1D). However, HIV-1p24 + human cells were not observed in the spleens of ART-treated mice, reflecting the undetectable plasma viral load (Fig. 1E). Overall, plasma viral load, blood CD4 + and CD8 + T cells profiles, and immunobiological tests together concluded sustained HIV-1 infection in vehicle-treated mice and reduced burden of viral infection in ART-treated mice.

### CEST MRI Detection of HIV-1-Induced Brain Glutamate Deficits and ART-Mediated Recovery

**Figure 2:**
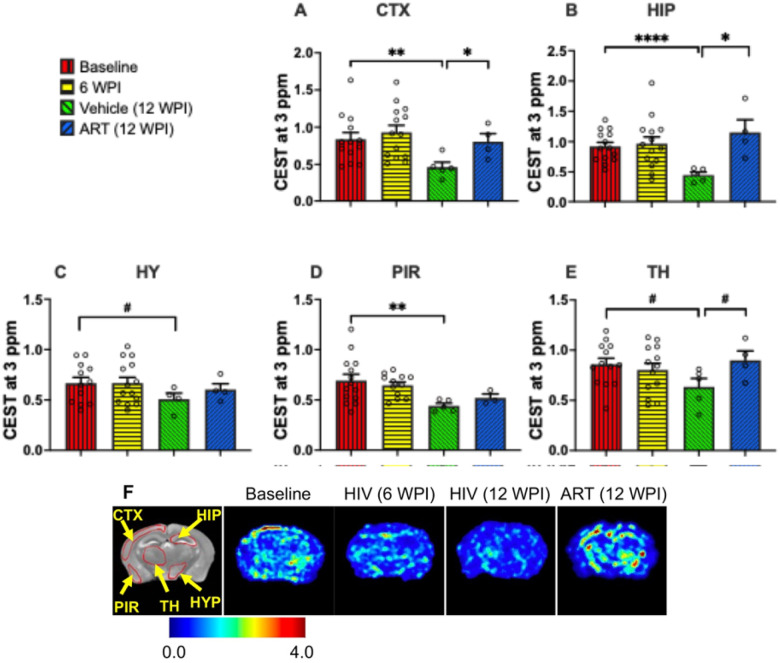
CEST contrast values at 3 ppm (associated with glutamate) in (**A**) cortex (CTX), (**B**) hippocampus (HIP), (**C**) hypothalamus (HY), (**D**) piriform cortex (PIR), and (**E**) thalamus (TH) regions. The experimental groups shown are baseline (red bars), HIV-infected at 6 WPI (yellow bars), HIV-infected at 12 WPI (green bars), and ART-treated at 12 WPI (blue bars). #: 0.05 < p < 0.1, *: 0.01 < p < 0.05, **: 0.001 < p < 0.01, ***: 0.0001 < p < 0.001, ****p < 0.0001. (**F**) 3 ppm contrast heatmaps of a representative mouse at baseline, a representative HIV-infected mouse at 6 WPI, a representative HIV-infected mouse at 12 WPI, and a representative ART-treated mouse at 12 WPI

CEST contrasts at 3 ppm are largely associated with glutamate. Thus, to determine the adverse effect of HIV-1 infection on glutamate levels and the benefits of ART treatment for reversing these changes, CEST contrast at 3 ppm was quantified in five brain regions (Fig. 2A–F). These included the cortex, hippocampus, hypothalamus, piriform cortex, and thalamus. The CEST contrast at 3 ppm for all brain regions was quantified as the area under the Lorentzian function at 3 ppm. At 6 WPI, no significant changes at 3 ppm contrast were observed in any of the analyzed brain regions compared to baseline (pre-infection, Week 0) levels. Such observation indicated that short duration of productive infection (6 weeks) does not affect 3 ppm-associated metabolites, particularly glutamate in humanized mice. However, at 12 WPI, significant reductions in 3 ppm contrast were noted in the cortex (p = 0.004), hippocampus (p < 0.0001), and piriform cortex (p = 0.002) of the vehicle-treated mice compared to those at baseline. In addition, trends of reduction were found in the hypothalamus (p = 0.09) and thalamus (p = 0.06) of these animals. Overall, reduction in CEST contrast at 3 ppm indicated decreases of glutamate levels due to prolonged infection. Interestingly, in ART-treated mice at 12 WPI, significantly higher CEST contrast at 3 ppm was measured in the cortex (p = 0.05) and hippocampus (p = 0.04) compared to in the respective regions of vehicle-treated mice. Trend of increased contrast was also observed in the thalamus (p = 0.06). Furthermore, comparable 3 ppm contrast was noted between ART-treated group at 12 WPI and the pre-infection baseline.

Heatmaps of 3 ppm contrast on group-representative mouse brains are shown in Fig. 2F. A T_2_-weighted image shows the anatomical references for the brain regions utilized to quantify 3 ppm contrasts. In the representative heatmap, decreased color intensity was observed in the brain of a vehicle-treated mouse at 12 WPI compared to that in the mouse brain from the baseline group, reflecting an HIV-1 infection-induced decrease in glutamate. Remarkably, higher color intensity was noted in the brains of ART-treated mice than that in the brains of the vehicle-treated mice at 12 WPI. In addition, similar color intensity was noted between baseline and ART-treated groups, signifying recovery of the 3 ppm contrast in the ART-treated mice. These data underscore the benefits of ART treatment in restoration of brain glutamate levels in HIV-1 infected mice.

### CEST MRI Detection of HIV-1-Induced Alterations in Brain Creatine and ART-Mediated Restoration

**Figure 3:**
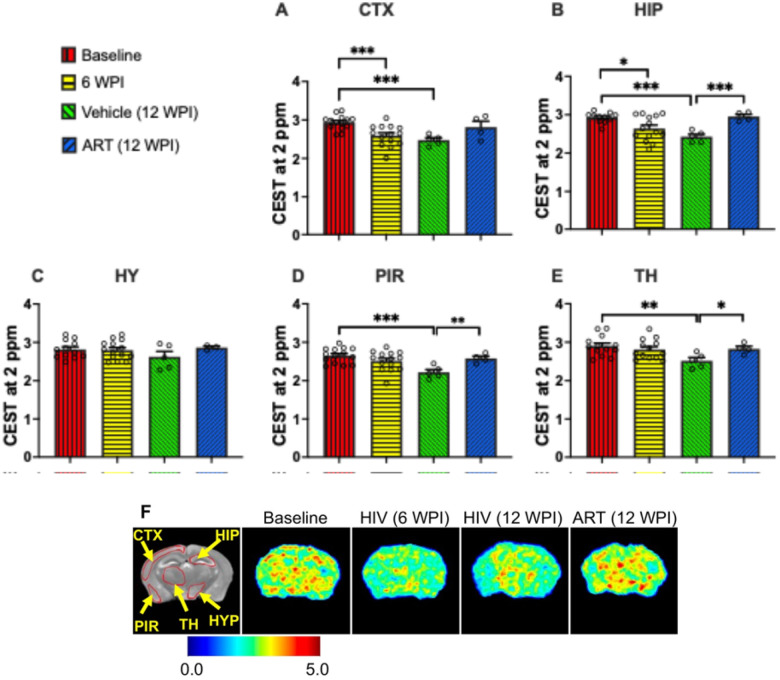
CEST contrast values at 2 ppm (associated with creatine) in (**A**) cortex (CTX), (**B**) hippocampus (HIP), (**C**) hypothalamus (HY), (**D**) piriform cortex (PIR), and (**E**) thalamus (TH) regions. The experimental groups shown are baseline (red bars), HIV-infected at 6 WPI (yellow bars), HIV-infected at 12 WPI (green bars), and ART-treated at 12 WPI (blue bars). #: 0.05 < p < 0.1, *: 0.01 < p < 0.05, **: 0.001 < p < 0.01, ***: 0.0001 < p < 0.001, ****p < 0.0001. (**F**) 2 ppm contrast heatmaps of a representative mouse at baseline, a representative HIV-infected mouse at 6 WPI, a representative HIV-infected mouse at 12 WPI, and a representative ART-treated mouse at 12 WPI

CEST contrasts at 2 ppm are linked to creatine levels. Therefore, to identify the effects of HIV-1 on creatine levels and whether ART treatment aids in their recovery, CEST contrast at 2 ppm was measured in the same five brain regions as 3 ppm measurements (Fig. 3A–F). In contrast to 3 ppm measurements, comparisons between baseline and 6 WPI data revealed a significant decrease in 2 ppm CEST contrast in the cortex (p = 0.0006) and hippocampus (p = 0.01). These findings suggested that short-term (6 weeks) HIV-1 infection can adversely affect creatine in humanized mice. At 12 weeks post-infection (WPI), vehicle-treated mice exhibited significant reductions in 2 ppm CEST contrast compared to baseline in the cortex (p = 0.0002), hippocampus (p = 0.0003), piriform cortex (p = 0.0009), and thalamus (p = 0.006). These observations at 2 ppm signified that HIV-1 infection leads to decline in creatine levels in humanized mice. Similar to 3 ppm observations, in ART-treated mice, significant elevated 2 ppm CEST contrast was found in the hippocampus (p = 0.0005), piriform cortex (p = 0.0062), and thalamus (p = 0.028) compared to those in vehicle-treated mice at 12 WPI. Comparable 2 ppm CEST contrast was observed between the pre-infected baseline and ART-treated groups, indicating recovery of brain creatine levels in HIV-1 infected mice following ART treatment.

Heatmaps of CEST contrast at 2 ppm contrast on group-representative mouse brains are shown in Fig. 3F. Heatmaps are reflective of quantitative data presented in Fig. 3A–E. Reduced color intensities were observed in the brains of mice at 6 WPI and in vehicle-treated mice at 12 WPI compared to baseline, indicating a decrease in brain creatine levels associated with HIV-1 infection. As expected, higher color intensity was seen in brains of ART-treated mice than the vehicle-treated mice, with similar color intensities observed between baseline and ART-treated mice. The CEST contrast at 2 ppm highlighted the efficacy of ART in recovery of brain creatine levels, which were impaired due to HIV-1 infection.

### CEST MRI Detection of HIV-1-Linked Turnover of Macromolecules

**Figure 4:**
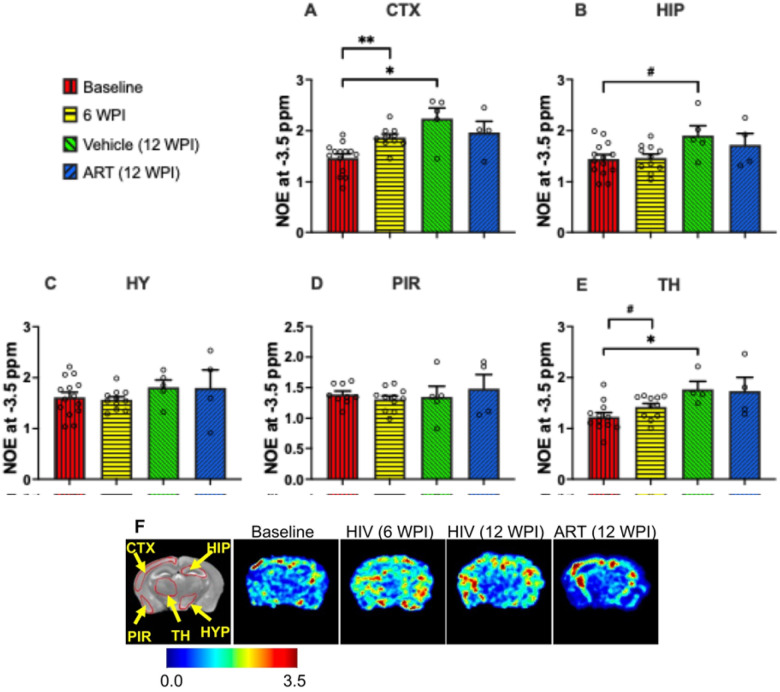
NOE contrast values (associated with membrane lipids) in (**A**) cortex (CTX), (**B**) hippocampus (HIP), (**C**) hypothalamus (HYP), (**D**) piriform cortex (PIR), and (**E**) thalamus (TH) regions. The experimental groups shown are baseline (red bars), HIV-infected at 6 WPI (yellow bars), HIV-infected at 12 WPI (green bars), and ART-treated at 12 WPI (blue bars). #: 0.05 < p < 0.1, *: 0.01 < p < 0.05, **: 0.001 < p < 0.01, ***: 0.0001 < p < 0.001, ****p < 0.0001. (**F**) NOE contrast heatmaps of a representative mouse at baseline, a representative HIV-infected mouse at 6 WPI, a representative HIV-infected mouse at 12 WPI, and a representative ART-treated mouse at 12 WPI.

NOE at −3.5 ppm associated with brain macromolecules (e.g., mobile proteins, lipids) were quantified (Fig. 4A–F). Analysis at −3.5 ppm revealed a significant increase in NOE signal in the cortex (p = 0.001) and a trend toward an increase in the thalamus (p = 0.09) at 6 WPI compared to baseline. By 12 WPI, NOE signals continued to rise in vehicle-treated mice, with significant increases observed in the cortex (p = 0.02) and thalamus (p = 0.03), along with a trend toward an increase in the hippocampus (p = 0.07), relative to baseline. No significant changes at −3.5 ppm were observed between ART- and vehicle-treated groups at 12 WPI. These results indicate that ART treatment did not induce a measurable impact on macromolecular recovery. Heatmaps of NOE on group-representative mouse brains are shown in Fig. 4F. Higher color intensity was observed in the brains of mice at 6 WPI, vehicle-treated mice at 12 WPI, and ART-treated mice at 12 WPI compared to baseline mice.

### HIV-1 Infection in Mouse Brains

**Figure 5:**
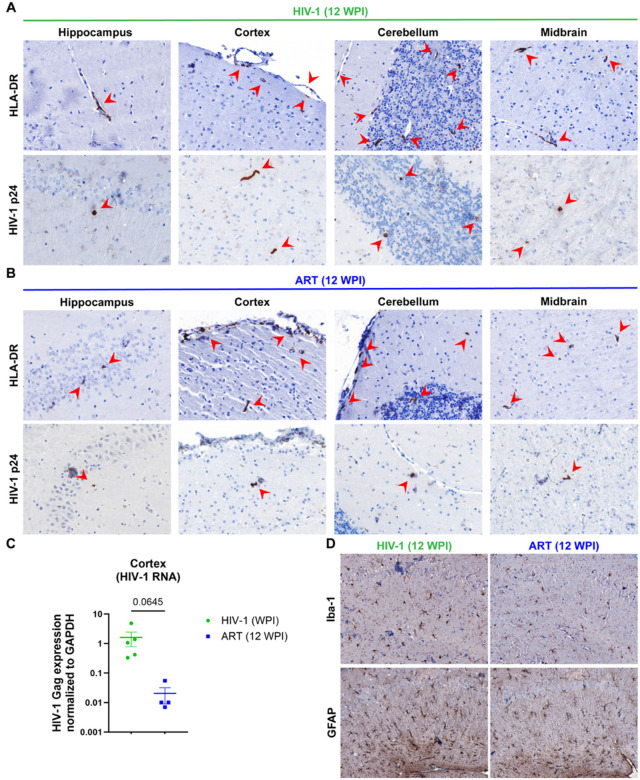
Immunohistochemical (IHC) analysis of brain regions from vehicle- and ART-treated HIV-infected humanized mouse models at 12 WPI. IHC staining for HLA-DR and HIV-1 p24 in the hippocampus, cortex, cerebellum, and midbrain from (**A**) vehicle-treated (**B**) ART-treated HIV-1 infected mice. Red arrowheads indicate areas of positive staining. (**C**) HIV-1 Gag expression in the cortex of vehicle-treated (green) and ART-treated (blue) mice. (**D**) Comparative IHC staining for Iba-1 (microglial activation marker) and GFAP (astrocyte activation marker) in vehicle- and ART-treated mouse brain hippocampal sections

To investigate brain infiltration of human cells, immunohistochemistry was performed at study termination (12 WPI). Brain sections were stained for human HLA-DR and HIV-1 p24 antigens. Human HLA-DR + cells infiltrated the brains of both vehicle- and ART-treated mice. These HLA-DR + cells were observed in several brain regions including hippocampus, cortex, cerebellum, and midbrain (Fig. 5A–B; indicated by red arrows, top row). HIV-1 p24 + human cells were observed in the same regions of mice from both groups (Fig. 5A–B; indicated by red arrows, bottom row). Although the number of human cells observed in brains were comparable for vehicle- and ART-treated mice, the number of HIV-1 p24 + cells were sparse in ART-treated mice compared to vehicle-treated mice. The level of viral infection in the cortex region was determined by real-time qPCR targeting HIV-1-Gag to quantify tissue viral RNA (Fig. 5C). Robust viral infection was observed in the cortical tissue of vehicle-treated mice, validating the sustained viral infection, whereas relatively lower infection was noted in ART-treated mice. Both immunohistology and qPCR tests confirmed the efficacy of ART-treatment in controlling brain viral infection. Indeed, viral infection was undetectable in the plasma and spleen in of ART-treated mice at 12 WPI. HIV-1 infection was detected in ART-treated brains, albeit at lower magnitudes.

Further, glial cell responses were assessed by glial fibrillary acidic protein (GFAP, astrocyte) and ionized calcium binding adaptor molecule-1 (Iba-1, microglia) staining (Fig. 5D). Hippocampus areas with hypertrophic astrocytes and morphological features of activated microglia were readily observed in vehicle-treated mice. Activated morphologies were observed, defined by increased cell body size and process formations for both astrocytes and microglia. However, activation features of both glial cells were reduced in ART-treated mice, potentially due to controlled viral replication. Although neuroinflammation was significantly reduced in ART-treated mice compared to vehicle-treated mice, glial cells with activated morphologies, to a lower extent, were still seen in brains of ART-treated mice. Overall, the efficacy of ART-treatment in reducing brain viral load and virus-linked glial cell activation in humanized mice was confirmed.

## DISCUSSION

This study elucidates significant neurometabolic perturbations in a humanized mouse model of HIV-1 infection. Notably, the study emphasizes the persistent adverse effects of infection on the neuroimmune system despite the potent efficacy of ART in controlling viral replication at undetectable levels. Novel CEST MRI investigation provides an understanding of HIV-1-associated metabolic impairments, potentially describing an underlying mechanism for HAND. These findings underscore the pivotal role of advanced neuroimaging techniques in unraveling the pathophysiological mechanisms of HAND and in monitoring therapeutic outcomes.

CEST contrast data at 3 ppm and 2 ppm offer robust evidence of HIV-1-induced neurometabolic disruptions and efficacy of ART in ameliorating these impairments. In vehicle-treated HIV-1 infected mice, significant reductions in contrasts at both 3 and 2 ppm were observed, indicating decreases in glutamate and creatine metabolites, respectively. Glutamate is a primary excitatory neurotransmitter. The reduction in brain glutamate levels demonstrates the glutamatergic reduction and neurodegenerative processes associated with chronic HIV-1 infection[69, 7]. In addition, the alterations in 2 ppm CEST contrast was attributed to creatine metabolism, reflecting cellular bioenergetic distress caused by HIV-1 infection[70, 54]. Further, flow cytometry and plasma viral RNA evaluations confirmed the ART-linked decrease in HIV-1 infection in the peripheral system and CNS. ART treatment led to recovery of contrasts at 3 and 2 ppm. Restoration of these contrasts following ART highlights its neuroprotective efficacy in preserving neuronal function. Furthermore, changes of NOE at −3.5 ppm revealed HIV-1-linked increase in macromolecular content, regardless of treatment status. Previous studies suggest that NOE arises from membrane lipids[52], indicating HIV-1-induced neuronal injury. Surprisingly, restoration of these macromolecules was not observed following ART treatment. The duration of the overall treatment scheme could explain this outcome, as CEST MRI and histological tests were completed following 6 weeks of treatment. Thus, recovery of glutamate and creatine could potentially relate to rapid recovery of neuronal and glial metabolism compared to membrane lipid dynamics. This observation is consistent with changing neuroinflammation (micro- and astrogliosis), which was mitigated in the ART-treated group compared to the HIV-infected vehicle-treated group, but not completely resolved to normal morphologies. Future investigations are needed to address whether macromolecules and inflammatory reactions return to normal or persist following long-term ART-treatment.

It must be noted that observed HIV-1-induced metabolic alterations were not specific to any brain region. These metabolic impairments were consistently seen in almost all assessed brain regions. Infiltration of human HIV-1 p24 + cells in these regions verified HIV-1-linked pathology, but provided no specificity to any one region. Infiltration of human immune cells (including those with HIV-1 infection) in all brain regions allows the humanized mouse model, in part, to reflect human neuroHIV pathobiology. The hallmarks of HIV-1 infection in humans (e.g. peripheral viral load and human CD4 + T-cell decline), are reflected in these humanized mice. In addition to our current data, metabolic encephalopathy was caused by viral infection, resulting in micro- and astro-gliosis, excitotoxicity, myelin injury, and neuronal injury, as seen previously in both humans and infected mice [71, 53]. Such spectrums of pathologies make the humanized mice a relevant model to study the HIV-1-induced neuropathology underlying clinical HAND manifestations and therapeutic outcomes of ART.

This study represents the first CEST MRI evaluation of neuroHIV, monitoring plausible biomarkers of HAND pathology[72, 73] and their changes following conventional ART treatment. The observed changes in CEST contrasts associated with glutamate, creatine, and NOE-linked macromolecules demonstrate the ability of CEST MRI to image HIV-linked neurocognitive impairment. Similarly, CEST detection of contrast restoration represents a novel technique for evaluating ART effectiveness non-invasively. The neurometabolic biomarkers identified herein could serve as critical tools for the early detection and longitudinal monitoring of HAND. Future research should address the limitations of the current study, including the restricted sample size, the absence of behavioral evaluation, and the lack of long-term follow-up data. Expanding the investigation to incorporate other advanced imaging techniques (e.g. diffusion tensor imaging [DTI] and magnetic resonance spectroscopy [MRS]) and exploring adjunctive therapies targeting residual macromolecular alterations will provide deeper insights into HAND pathogenesis and therapeutic strategies.

## CONCLUSION

This study emphasizes the transformative application of MRI in elucidating HIV-1-associated neurometabolic alterations. Through advanced applications of CEST MRI, this research highlights ART’s neuroprotective effects on the neural metabolic profile while revealing persistent macromolecular changes, such as those associated with membrane lipids. These findings underscore the pivotal role of MRI as a non-invasive tool for diagnosing and monitoring HAND, offering unparalleled insights into both metabolic disruptions and therapeutic outcomes. Collectively, these results advocate for the broader adoption of MRI in neuroHIV research to enhance diagnostic accuracy, therapeutic efficacy, and overall management of HAND, ultimately improving the quality of life for PLWH.

## Supplementary Files

This is a list of supplementary files associated with this preprint. Click to download.

- GatinglayoutforManuscriptCaptioned.pdf

## Data Availability

The datasets generated during and/or analyzed during the current study are not publicly available, but are available from the corresponding author upon request.
